# Use of small rodents for the surveillance of agents and vectors of tick-borne zoonoses in the northern Apennines, Italy

**DOI:** 10.1186/1756-3305-7-S1-O36

**Published:** 2014-04-01

**Authors:** E Martello, A Mannelli, C Ragagli, M Selmi, C Ambrogi, E Grego, LA Ceballos, MC Stella, L Tomassone

**Affiliations:** 1 Dipartimento di Scienze Veterinarie, University of Torino, Grugliasco, Italy; 2 Ufficio Territoriale per la Biodiversità, Corpo Forestale dello Stato, Lucca, Italy; 3 Osservatorio Permanente per Patologie a trasmissione Vettoriale, ASL2, Lucca, Italy

## 

Vector borne zoonoses are emerging threats in Europe. Data collection from animals may be useful to evaluate their occurrence and intensity of transmission, and to detect their introduction into previously free geographic areas. Indeed, vertebrates serve as hosts for pathogens and for arthropod vectors, although their ecological role can vary according to the diseases.

Data collection on small rodents was used to study the eco-epidemiology of two tick-borne pathogens, *Rickettsia slovaca *(agent of tick-borne lymphadenopathy, transmitted by *Dermacentor marginatus*) and *Borrelia burgdorferi *sensu lato (agent of Lyme Borreliosis, transmitted by *Ixodes ricinus*), in the Apennine mountains, Tuscany (Italy), where human cases of tick-borne diseases were reported. Small rodents are preferential hosts for the immature stages of the two tick vectors and are involved in the transmission cycle of both diseases.

In the summers from 2009 to 2012, we live trapped *Apodemus *spp. and *Myodes glareolus *from 1100 to 1650 m above the sea level (a.s.l.). Rodents were found infested by immature *I. ricinus *and *D. marginatus*. The monthly activities of these two tick species on the same hosts were different, reflecting differences in their life cycles. Although few individuals were co-infested, both tick species tended to aggregate on the same *Apodemus *spp. males. *R. slovaca *and *B. burgdorferi *s.l. were detected in rodent ear biopsies and attached ticks up to 1650 m a.s.l. In our study area, rodents might play a role as amplifiers of *R. slovaca *infection; even in the absence of the host's systemic infection, tick aggregation on the same individuals might favour the transmission of the pathogen through co-feeding. While *D. marginatus *had been found at the same location in studies carried out in 1994, *I.ricinus *was very rare or absent. Data collection on small rodents thus highlighted the recent range expansion of *I. ricinus *and *B. burgdorferi *s.l. in a previously unoccupied area. Major land use changes, the increased abundance of wildlife populations, as well as a general climate warming in the Mediterranean area, might be interacting factors affecting the altitudinal range expansion of *I. ricinus *in the Northern Apennines, and in Italy in general.

